# Emergence of Vaccine-derived Polioviruses, Democratic Republic of Congo, 2004–2011

**DOI:** 10.3201/eid1910.130028

**Published:** 2013-10

**Authors:** Nicksy Gumede, Olivia Lentsoane, Cara C. Burns, Mark Pallansch, Esther de Gourville, Riziki Yogolelo, Jean Jacques Muyembe-Tamfum, Adrian Puren, Barry D. Schoub, Marietjie Venter

**Affiliations:** National Institute for Communicable Diseases, Johannesburg, South Africa (N. Gumede, O. Lentsoane, A. Puren, B.D. Schoub, M. Venter);; University of Witwatersrand, Johannesburg (N. Gumede, B.D. Schoub);; University of Pretoria, Pretoria, South Africa (M. Venter);; Centers for Disease Control and Prevention, Atlanta, Georgia, USA (C.C. Burns, M. Pallansch);; World Health Organization, Geneva, Switzerland (E. de Gourville);; National Institute for Biomedical Research, Kinshasa/Gombe, Democratic Republic of Congo (R. Yogolelo, J.J. Muyembe-Tamfum)

**Keywords:** vaccine-derived poliovirus, poliovirus, circulating VDPV, viruses, Democratic Republic of Congo, DRC

## Abstract

Polioviruses isolated from 70 acute flaccid paralysis patients from the Democratic Republic of Congo (DRC) during 2004–2011 were characterized and found to be vaccine-derived type 2 polioviruses (VDPV2s). Partial genomic sequencing of the isolates revealed nucleotide sequence divergence of up to 3.5% in the viral protein 1 capsid region of the viral genome relative to the Sabin vaccine strain. Genetic analysis identified at least 7 circulating lineages localized to specific geographic regions. Multiple independent events of VDPV2 emergence occurred throughout DRC during this 7-year period. During 2010–2011, VDPV2 circulation in eastern DRC occurred in an area distinct from that of wild poliovirus circulation, whereas VDPV2 circulation in the southwestern part of DRC (in Kasai Occidental) occurred within the larger region of wild poliovirus circulation.

## Abstract 2

TOC summary: These viruses can emerge independently where immunity to poliovirus is inadequate.

Indigenous wild-type poliovirus (WPV) remains endemic to 3 countries: Nigeria, Afghanistan, and Pakistan (*1*). Poliovirus (PV) circulation has been sustained in several African countries after importation from a PV-endemic country, resulting in reestablished virus transmission. Worldwide, the number of cases decreased by 50% from 2010 to 2011 (*2*). In developing countries, live attenuated oral PV vaccine (OPV) is still the vaccine of choice. However, the virus can revert to virulence during OPV replication in humans, resulting in person-to-person spread and circulation of vaccine-derived PVs in areas with low rates of vaccination coverage (*3*). Substantial sequence drift occurs in circulating VDPVs (>1% nt difference in types 1 and 3, >0.6% nt difference in type 2), indicating prolonged replication of the vaccine strain in human populations and consequent changes in phenotypic properties of neurovirulence and transmissibility (*3*,*4*). Poliomyelitis outbreaks associated with circulating VDPVs have been reported in several countries, including Egypt (1982–1993), Haiti (2000–2001), Dominican Republic (2000–2001), Philippines (2001), Madagascar (2002 and 2005), China (2004), Cambodia (2005–2006), Indonesia (2005), and Nigeria 2005–2010 (*4**–**11*). As a result of accumulating evidence about the emergence and spread of circulating VDPVs, there are plans to stop using OPV and synchronously implement more widespread use of inactivated polio vaccine (*12*–*15*). A better understanding of VDPV persistence and circulation is crucial for deciding when and how to stop vaccination with OPV after WPVs have been eradicated (*16*–*18*).

In early 2001, indigenous WPVs were eliminated from DRC, but starting in 2006 and continuing through 2011, WPV was imported into DRC from Angola several times. During 2010−2011 in DRC, 2 genetic clusters of the Southeast Asian PV1 genotype circulated; this genotype was imported twice from India to Angola and subsequently to DRC. Since December 2011, no cases of infection with WPV have been detected in DRC (*19*).

Monovalent OPV types 1 and 3 and bivalent OPV effectively induce immunity because of a lack of interference by the type 2 component (*20*). Reliance on monovalent OPV1, monovalent OPV3, and bivalent OPV in supplemental vaccination activities has contributed to the emergence of VDPV2. These alternative OPV formulations are more effective than trivalent OPV at inducing higher levels of population immunity to WPV1 and WPV3 because there is no interference from the type 2 OPV strain. No type 2–specific immunity is induced. To maintain population immunity to type 2 PV, the World Health Organization (WHO) recommends 2 doses of trivalent OPV each year. 

We describe the genetic characterization of circulating VDPV2 associated with outbreaks in DRC. During 2004–2011, the same time that extensive circulating VDPV transmission occurred in Nigeria, VDPV2 excretion was found for 70 children with acute flaccid paralysis (*10*,*11*). The close genetic relationships among many of the viruses provide evidence for circulation in several regions of DRC.

## Materials and Methods

### Viruses

National authorities in DRC submitted fecal specimens from patients with acute flaccid paralysis to the National Institute for Biomedical Research, Kinshasa, for PV isolation by standard methods recommended by WHO. To determine serotype of PV isolates and whether the virus was wild or related to vaccine strains (known as intratypic differentiation), isolates were forwarded to the National Institute for Communicable Diseases in South Africa for characterization by PCR, ELISA, and partial genomic sequencing (*21*). The original fecal specimens from which these isolates were obtained were also sent to the National Institute for Communicable Diseases for confirmation of virus isolation results by methods recommended by WHO (*22*).

### Intratypic Differentiation 

Through use of PCR (*21*–*23*) and ELISA, as recommended by WHO, PV isolates were determined to be Sabin-like or non–Sabin-like strains (*23*–*26*). All serotyped PVs or virus isolates that had shown cytopathic effect in L20B cells were tested by using a reverse transcription PCR (RT-PCR) kit supplied by the Centers for Disease Control and Prevention (Atlanta, GA, USA), which included separate reactions with primers for panenterovirus; panpoliovirus; PV serotypes 1, 2, and 3 (*23*,*24*); and multiplexed primers for Sabin type 1, 2, and 3 PVs (*25*). The amplicons were separated on 10% polyacrylamide gels and visualized after staining with ethidium bromide. Additionally, serotyped PV monotypes were analyzed by using an ELISA developed by the National Institute for Public Health and the Environment (Bilthoven, the Netherlands) and identified as Sabin-like or non–Sabin-like strains by using specific cross-adsorbed antiserum (*21*). From 2007 on, a real-time PCR was used for intratypic differentiation of untyped L20B isolates with a positive cytopathic effect. Another real-time PCR was used for retrospective VDPV screening of Sabin strains reported during 2004−2006 and for prospective screening of untyped strains isolated from 2009 on (*27*,*28*).

### Sequencing

RNA was extracted from 140 µL of cell culture supernatant by using a QIAamp viral RNA extraction kit (QIAGEN GmbH, Hilden, Germany) according to the manufacturer’s recommendations. RT-PCR was performed in a single step, as described (*29*). Briefly, the extracted RNA (10 µL) was added to 90 µL of the amplification mixture containing 10 µL standard 10× reaction buffer, 100 µM of each dNTP (Roche Diagnostics GmbH, Mannheim, Germany), 10 mM dithiothreitol, 80 pmol of each primer (Q8 and Y7), 20 U of placental RNase inhibitor (Roche Diagnostics GmbH), 12.5 U of avian myeloblastosis virus reverse transcriptase (Roche Diagnostics GmbH), and 5U of Taq DNA polymerase (Roche Diagnostics GmbH). RT was conducted at 42°C for 60 min in a GeneAmp 9700 thermocycler (Applied Biosystems, Foster City, CA, USA) followed by denaturation at 95°C for 3 min. Amplification consisted of 30 cycles (95°C for 30 sec, 42°C for 30 sec, and 60°C for 2 min). The amplicons were visualized by ethidium bromide staining after electrophoresis on 1.5% agarose gels as described (*26*).

Before sequencing, the RT-PCR products were purified with the QIAquick PCR purification kit (QIAGEN). The complete viral protein (VP) 1 gene (nt 2480–3385) was sequenced as described (*29*,*30*) by cycle sequencing with the BigDye Terminator version 3.1 Cycle Sequencing Kit (*31*) (Applied Biosystems). The DNA sequence was determined by using the ABI 3100 Genetic Analyzer, version 3.1 (Applied Biosystems). Raw data files were imported into the Sequencher software package version 4.1.4 (http://avaxho.mc/software/soaftware_type/scientific/others/sequencher_414.htm) for assembly and editing, and consensus sequence files were produced. 

### Sequence Analysis

According to WHO guidelines, Sabin viruses that differ from Sabin 2 by >5 nt in the VP1 coding region are classified as VDPVs (*4*). To determine VP1 genetic diversity, we compared all complete VP1 sequences of VDPV2 isolates from patients with acute flaccid paralysis and their contacts with the sequence of the Sabin type 2 OPV reference strain (GenBank accession no. AY184220). Evolutionary distances were computed by using the Kimura 2-parameter method (*32*) and the neighbor-joining method (*33*,*34*). Phylogenetic trees were constructed by using MEGA5 (*35*) with 500 bootstrap replicates. Bootstrap values >80 (out of 100) are indicated on the tree.

The alignments (nucleotide and translated amino acid sequences) were analyzed by using GeneDoc version 2.6001 (www.psc.edu/biomed/genedoc) and MEGA5 (*35*) to identify specific mutations and positive selection. Assignment of isolates to independent VDPV2 emergences and lineage groups was based on pairwise VP1 capsid region sequence differences within and between lineage groups. Complete capsid region sequence differences were used when there was uncertainty about relationships that had been based on the VP1 region; complete genome sequences were used to identify possible recombination junctions (data not shown).

## Results

### Circulating VDPVs 

In total, just over 600 viral isolates obtained from DRC during 2004–2011 were tested by RT-PCR by using panenterovirus; panpoliovirus; serotype-specific; and Sabin type 1, 2, and 3 virus–specific primers. Isolates received before implementation of diagnostic real-time PCR were further tested by ELISA, and both techniques identified the isolates as Sabin-like PVs. All isolates were further screened by using real-time RT-PCR, as has been implemented in the Global Polio Laboratory Network, which included a screen for VDPVs (*9*). The genetic variability of virus isolates was further investigated by performing nucleotide sequence analysis of the VP1 capsid region of isolates that were identified as possible VDPVs by the VDPV screening assay. Partial genomic sequencing confirmed that for 70 cases, VP1 sequence was <99.3% identical to that of the parental Sabin serotype 2 strain (i.e., >6 nt substitutions), and the isolates were classified as VDPVs. In all isolates, VP1 amino acid changes associated with the reversion to virulence (*3*) were identified at position 143.

**Figure1:**
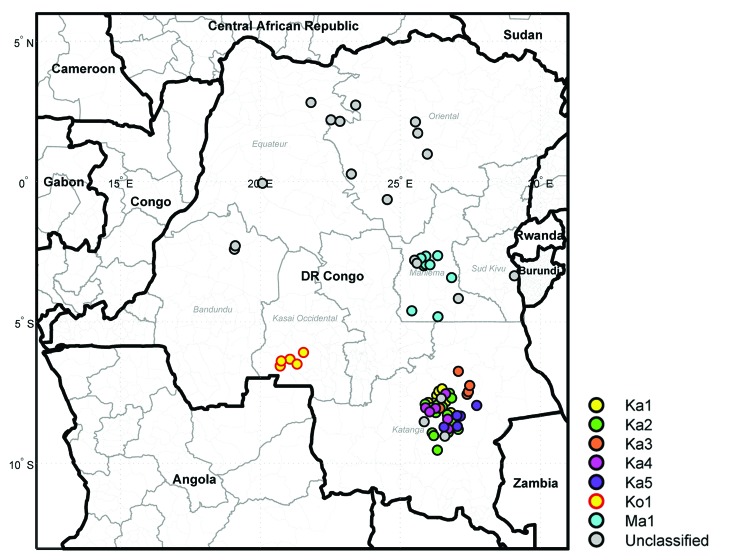
Geographic distribution of vaccine-derived polivirus type 2 from patients from the Democratic Republic of Congo (DR Congo). Viruses are represented by circles colored by lineage. Viruses that are not assigned to a lineage are categorized as unclassified.

**Figure 2:**
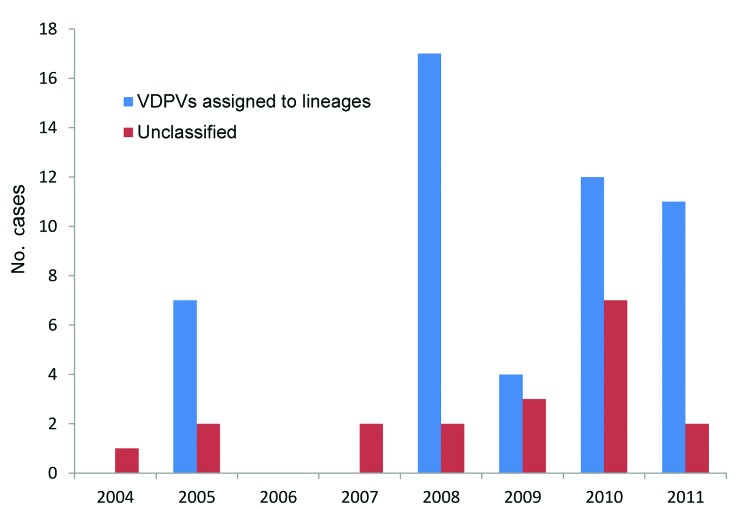
Yearly incidence of vaccine-derived polivirus type 2, Democratic Republic of Congo, 2004–2011. The total number of cases associated with vaccine-derived polivirus type 2 is graphed for each year according to date of onset of paralysis. Viruses that are not assigned to a lineage are categorized as unclassified. VDVPs, vaccine-derived PVs.

During 2004–2011, VDPV2s were isolated from fecal specimens from patients with acute flaccid paralysis in several regions of DRC (Technical Appendix). The first case occurred in Maniema Province, in Kindu District, on October 13, 2004. For the other provinces, 40 cases occurred in Katanga, 12 in Maniema, 6 in Orientale, 5 in Kasai Occidental, 4 in Équateur, 2 in Bandundu, and 1 in Sud Kivu (Figures 1,2). During 2010–2011, a total of 193 cases of WPV1 were also reported in DRC; some of the geographic areas where VDPVs were found overlapped. During this time, ongoing reestablished transmission of WPV1 in the eastern part of the country occurred in northeastern Katanga, whereas VDPVs were found in central Katanga. In 2010, in Kasai Occidental, southwestern DRC, a different genetic cluster of WPV1 was introduced from Angola and the Republic of Congo. The extensive area of WPV1 circulation overlapped with the more restricted area of circulation of VDPVs in Kasai Occidental (*19*).

### Phylogeny of the VP1 Region of the VDPVs

**Figure 3:**
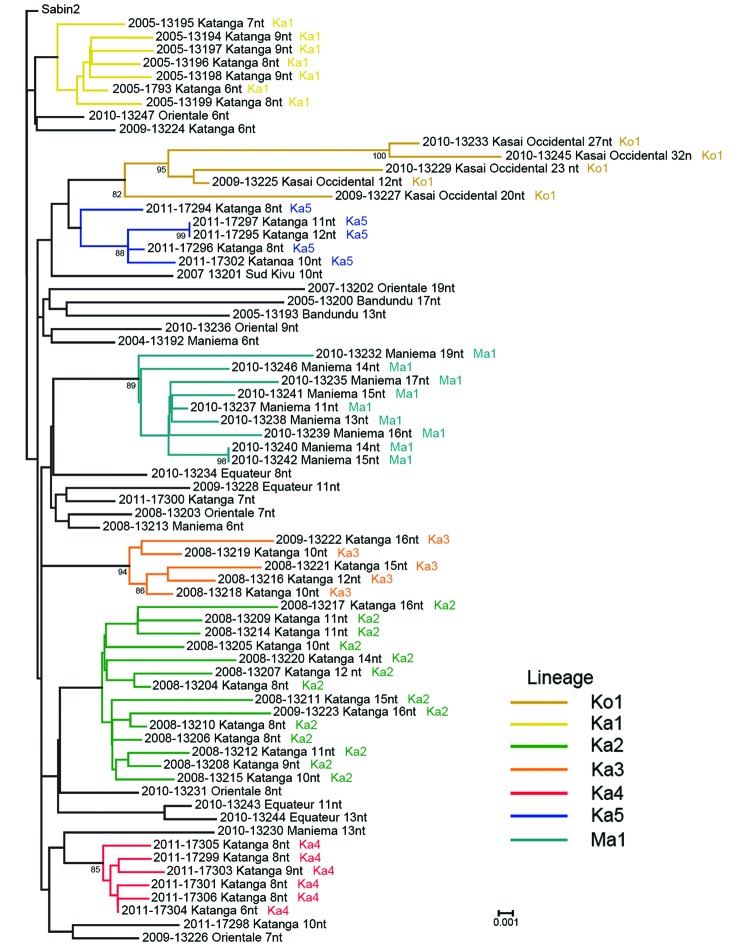
Viral protein phylogenetic relationships among vaccine-derived poliviruses isolated from patients with acute flaccid paralysis, Democratic Republic of Congo, 2004–2011. The tree was rooted to the Sabin type 2 poliovirus sequence. The year of onset of paralysis is indicated at the beginning of each virus name, followed by a 5-digit identifier and the province of the case-patient. The numbers of nucleotide differences from the Sabin 2 prototype viral protein 1 sequence are indicated, followed by the name of the lineage. Bootstrap values >80 are indicated on the tree. Scale bar indicates nucleotide substitutions per site.

Emergence of VDPV2 was first detected in Maniema Province in October 2004. No other related viruses were detected (Figure 3). Emergence of VDPV was next detected in Katanga in July 2005 (Figure 3). Four additional polio cases with onset during the same month were identified in Katanga, and 2 others followed in August. Viruses from the patients with polio onset in July and August were closely related to each other, forming lineage Ka1, which circulated in the district of Kinkondja in Katanga. Circulation of this lineage apparently stopped abruptly in 2005; no additional related viruses were detected in later years. The other 4 viruses isolated during 2005 and 2007 represented independent VDPV emergence events in 3 provinces (Bandundu, Sud-Kivu, and Orientale).

In 2008, two concurrent VDPV2 outbreaks (lineages Ka2 and Ka3) were detected in Katanga (Figure 3). Both outbreaks continued into 2009 in central Katanga. Also in 2008, two individual VDPV emergence events were detected in Maniema and Orientale.

In 2009, an outbreak in Kasai Occidental was detected and continued into 2010 (Ko1 lineage). In June 2010, an outbreak was detected in Maniema Province in several districts (Ma1 lineage); the last case occurred in October 2010. In addition, in 2009 and 2010, individual VDPVs representing independent emergence events were detected in Equateur, Maniema, Oriental, and Katanga Provinces.

In late 2011, several VDPVs were detected in Katanga; 4 independent events of VDPV emergence were observed, 2 of which formed lineages consisting of 5 or 6 VDPVs with 0.6%–1.2% nt difference from Sabin 2 (Ka4 and Ka5). During the 7-year period, the Katanga outbreaks accounted for 40 VDPVs, which is more than half of the total number of VDPVs detected in DRC.

## Discussion

We isolated VDPVs from fecal specimens from 70 patients with acute flaccid paralysis in DRC during 2004–2011. Sequence analysis of the VP1 coding region of these viruses showed that 51 isolates represented 7 circulating lineages of circulating VDPVs, and 19 others represented independent emergence events. The detection of several distinct circulating VDPV lineages in DRC reinforces previous observations that circulating VDPVs can emerge independently in locations where immunity to PV is inadequate, such as northern Nigeria, (*11**,**36*) where 24 emergence events were observed (*11*,*37*). Favorable conditions for VDPV emergence existed throughout much of DRC.

More than half of the VDPVs identified were associated with cases from Katanga and were isolated during 2005, 2008, 2009, and 2011. WPV1 circulated in Katanga in 2010 and 2011 in districts other than those with circulating VDPV. Social mobilization efforts to increase vaccination acceptance are ongoing in Katanga because of a relatively high frequency of parents who refuse to have their children vaccinated, some for religious reasons (*38*).

The relative risks for paralysis from WPV1 and VDPV2 seem to be driven primarily by the particular serotype; the disease-to-infection ratio is higher for type 1 than for type 2. In a study of acute flaccid paralysis patients in Nigeria, clinical characteristics of cases associated with VDPV infection were similar to those associated with cases of WPV1 infection (*11*). Similar studies have not been performed for DRC.

Vaccination activities conducted in response to the outbreaks in 2008 and 2009 probably contributed to the disruption of VDPV and WPV transmission in 2010. In response to the VDPV2 outbreak, mass vaccination campaigns were performed in areas of Maniema, Kasai Occidental, and Katanga. Trivalent OPV was used in these campaigns. Monovalent OPV1 and bivalent OPV were used to control circulating WPV1. After circulation of lineages Ka1–Ka3 had stopped, circulating VDPV was again detected in Katanga in late 2011 (Ka4 and Ka5). As in 2008, simultaneous emergence of >1 VDPV lineage occurred in Katanga. Several VDPVs were detected in 2012 in Katanga; their characterization is ongoing (Qi Chen et al., unpub. data).

Events that occurred early in the circulating VDPV emergence pathway were observed for some lineages. OPV strains that differ from Sabin strains by 1–5 nt are being routinely identified by the sensitive VDPV screening assay in use in Global Polio Laboratory Network laboratories, and these minimally drifted Sabin-related viruses can occasionally be linked to circulating VDPVs. Figure 3 includes several isolates that differ from the Sabin 2 prototype VP1 sequence by 6 nt. A Katanga isolate that differed from the Sabin strain by 3 nt was genetically linked to a Katanga VDPV (13224) that differed from the Sabin strain by 6 nt (data not shown).

Before use of the sensitive VDPV screening assay was implemented, events that occurred early in the circulating VDPV emergence pathway were not observed. In the circulating VDPV type 1 outbreak in Hispaniola, early events were missed because of delayed recognition of the outbreak. The study of early events in circulating VDPV emergence is yielding valuable information for use in planning the endgame strategy for polio eradication. It now seems necessary to synchronize the phase-out of OPV to minimize the emergence of circulating VDPVs, particularly circulating VDPV2. Emergence of VDPVs from the Sabin OPV2 strain has been much more frequent than emergence of VDPVs from OPV1 and OPV3. Discontinuation of the type 2 component of OPV is being considered as a strategy for reducing the frequency of circulating VDPV2 emergence (*12*).

In this study, each DRC viral lineage was found to be restricted to a limited geographic region. This finding is consistent with the relative lack of long-range transportation networks across DRC and the low rate of routine vaccination coverage throughout DRC, which results in gaps in immunity to type 2 PV. Without an extensively developed highway system, individual geographic regions tend to be isolated and human population movement is restricted. With limited movement of people across long distances, virus transmission is restricted. The situation in DRC contrasts with that in northern Nigeria, where large-scale population movement occurs between the west and east across the northern states. Wild and circulating VDPV lineages spread widely throughout the northern states of Nigeria (*11*).

The circulating VDPVs detected in DRC had recovered the 2 most important biological properties of WPVs, namely the capacity to cause paralytic disease in humans and the capacity for continuous person-to-person transmission. The origin of circulating VDPV2 in DRC was probably the result of low population immunity to PV because of a combination of low vaccination coverage in some communities (*39*) and the prior elimination of the indigenous WPV of the same serotype (*20*). Because WPV2 has not circulated anywhere globally since 1999, immunity to PV type 2 is not being stimulated through virus circulation. Routine vaccination coverage is low in many regions of DRC; estimated OPV3 coverage was 73% (*40*). Additional factors that probably facilitated circulation of VDPVs in some communities were poor hygiene, inadequate sanitation, and tropical climate. Because similar conditions exist elsewhere, ongoing high-quality surveillance will be essential for eliminating polio in Africa. The need for vigilance was confirmed by the detection of circulating VDPV2 and circulating VDPV3 in Ethiopia (Gumede et al., unpub. data), circulating VDPV2 in Chad, and circulating VDPV2 in Somalia (*20*). The occurrence of WPV outbreaks in DRC emphasizes the need to maintain high vaccine coverage and acute flaccid paralysis surveillance to minimize the risk for emergence of VDPVs or circulation of imported WPVs.

Technical Appendix Vaccine-derived polioviruses isolated in the Democratic Republic of Congo, 2004– 2011.
