# Supplementary material for: Emergence of Vaccine-derived Polioviruses, Democratic Republic of Congo, 2004–2011
Source: Emerg Infect Dis. 2013 Oct;19(10):1583–9. doi: 10.3201/eid1910.130028 (PMC3810735; doi:10.3201/eid1910.130028)
Supplement: Technical Appendix — Vaccine-derived polioviruses isolated in the Democratic Republic of Congo, 2004– 2011. [file 13-0028-Techapp-s1.pdf]

Article DOI: http://dx.doi.org/10.3201/eid1910.130028

Emergence of Vaccine-Derived Polioviruses, Democratic Republic of Congo, 2004–2011

Technical Appendix

Technical Appendix Table. Vaccine-derived polioviruses isolated in the Democratic Republic of Congo, 2004– 2011

Isolate	Province	District	Date of onset	GenBank accession no.	Lineage
1793	Kat	Kinkondja	7/25/2005	JQ691358	Ka1
13192	Man	Kindu	10/13/2004	JQ691357	-
13193	Bdd	Inongo	1/1/2005	JQ691360	-
13194	Kat	Malemba-Nkulu	1/1/2005	JQ691361	Ka1
13195	Kat	Kinkondja	7/8/2005	JQ691362	Ka1
13196	Kat	Kinkondja	7/20/2005	JQ691363	Ka1
13197	Kat	Kinkondja	7/28/2005	JQ691364	Ka1
13198	Kat	Kinkondja	8/23/2005	JQ691365	Ka1
13199	Kat	Kinkondja	8/31/2005	JQ691366	Ka1
13200	Bdd	Inongo	9/9/2005	JQ691367	-
13201	Skv	Uvira	3/16/2007	JQ691368	-
13202	Ort	Bafwasende	12/15/2007	JQ691369	-
13203	Ort	Yaleko	1/19/2008	JQ691370	-
13204	Kat	Kinkondja	1/19/2008	JQ691371	Ka2
13205	Kat	Kinkondja	2/11/2008	JQ691372	Ka2
13206	Kat	Kinkondja	3/22/2008	JQ691373	Ka2
13207	Kat	Kinkondja	3/27/2008	JQ691374	Ka2
13208	Kat	Butumba	6/12/2008	JQ691375	Ka2
13209	Kat	Kinkondja	6/17/2008	JQ691376	Ka2
13210	Kat	Butumba	5/15/2008	JQ691377	Ka2
13211	Kat	Bukama	4/12/2008	JQ691378	Ka2
13212	Kat	Mukanga	6/12/2008	JQ691379	Ka2
13213	Man	Kindu	6/7/2008	JQ691380	-
13214	Kat	Mukanga	7/6/2008	JQ691381	Ka2
13215	Kat	Malemba-Nkulu	7/9/2008	JQ691382	Ka2
13216	Kat	Mulongo	7/29/2008	JQ691383	Ka3
13217	Kat	Malemba-Nkulu	8/29/2008	JQ691384	Ka2
13218	Kat	Malemba-Nkulu	9/25/2008	JQ691385	Ka3
13219	Kat	Mulongo	10/8/2008	JQ691386	Ka3
13220	Kat	Kinkondja	10/19/2008	JQ691387	Ka2
13221	Kat	Manono	12/15/2008	JQ691388	Ka3
13222	Kat	Manono	2/25/2009	JQ691389	Ka3
13223	Kat	Butumba	3/2/2009	JQ691390	Ka2
13224	Kat	Kabondo-Dianda	6/26/2009	JQ691391	-
13225	Koc	Kamuesha	8/9/2009	JQ691392	Ko1
13226	Ort	Banalia	12/15/2009	JQ691393	-
13227	Koc	Luebo	12/27/2009	JQ691394	Ko1
13228	Eqt	Yambuku	12/31/2009	JQ691395	-
13229	Koc	Kalonda - Ouest	2/24/2010	JQ691396	Ko1
13230	Man	Salamabila	3/3/2010	JQ691397	-
13231	Ort	Aketi	3/21/2010	JQ691398	-

Isolate	Province	District	Date of onset	GenBank accession no.	Lineage
13232	Man	Kibombo	4/20/2010	JQ691399	Ma1
13233	Koc	Kamuesha	4/22/2010	JQ691400	Ko1
13234	Eqt	Monieka	5/2/2010	JQ691401	-
13235	Man	Kindu	6/19/2010	JQ691402	Ma1
13236	Ort	Banalia	6/25/2010	JQ691403	-
13237	Man	Kindu	6/30/2010	JQ691404	Ma1
13238	Man	Samba	7/6/2010	JQ691405	Ma1
13239	Man	Alunguli	8/7/2010	JQ691406	Ma1
13240	Man	Kindu	8/13/2010	JQ691407	Ma1
13241	Man	Kailo	8/17/2010	JQ691408	Ma1
13242	Man	Kindu	8/22/2010	JQ691409	Ma1
13243	Eqt	Bumba	8/29/2010	JQ691410	-
13244	Eqt	Bumba	9/6/2010	JQ691411	-
13245	Koc	Tshikapa	9/24/2010	JQ691412	Ko1
13246	Man	Kampene	10/13/2010	JQ691413	Ma1
13247	Ort	Yahuma	3/1/2010	JQ691359	-
17294	Kat	Mukanga	10/17/11	JX424836	Ka5
17295	Kat	Mukanga	10/21/11	JX424835	Ka5
17296	Kat	Mukanga	11/5/11	JX424834	Ka5
17297	Kat	Mukanga	11/10/11	JX424833	Ka5
17298	Kat	Kinkondja	11/26/11	JX424832	-
17299	Kat	Kinkondja	11/10/11	JX424831	Ka4
17300	Kat	Butumba	12/20/11	JX424830	-
17301	Kat	Butumba	12/3/11	JX424827	Ka4
17302	Kat	Butumba	12/17/11	JX424829	Ka5
17303	Kat	Kinkondja	12/15/11	JX424826	Ka4
17304	Kat	Kinkondja	11/4/11	JX424825	Ka4
17305	Kat	Kinkondja	12/29/11	JX424824	Ka4
17306	Kat	Malemba-Nkulu	12/30/11	JX424828	Ka4
